# Impact of nocturnal oxygen supplementation on sleep quality and daytime fatigue among interstitial lung disease (ILD) patients

**DOI:** 10.6026/973206300222457

**Published:** 2026-04-30

**Authors:** Mriganka Madhav Misra, Deepika Patel, Ashwin Songara, Anindita Bhagawati

**Affiliations:** 1 Department of Pulmonary Medicine, Assam Medical College, Dibrugarh, Assam, India

**Keywords:** Interstitial lung disease (ILD), nocturnal hypoxemia, polysomnography, oxygen therapy, sleep quality, quality of life

## Abstract

A collection of more than 300 conditions known as interstitial lung diseases (ILDs) limit oxygen delivery by generating persistent inflammation and irreversible lung tissue scarring, or fibrosis. There is progressive impairment of gas exchange and are frequently complicate by nocturnal hypoxemia. It remains under-recognized despite its significant impact on sleep quality and daytime functioning. Therefore, it is of interest to report the effect of nocturnal oxygen supplementation on sleep quality and daytime fatigue in patients with interstitial lung disease. Total included 228 participants were randomized into two groups: nocturnal oxygen supplementation (n=114) and placebo (sham oxygen) (n=114). Sleep parameters were assessed using overnight monitoring. The Pittsburgh Sleep Quality Index (PSQI), while daytime fatigue and sleepiness were evaluated using validated scales. Statistical analysis was performed using appropriate inferential tests, with p < 0.05 considered significant. Nocturnal oxygen supplementation significantly improves both objective and subjective sleep parameters while reducing daytime fatigue in ILD patients.

## Background:

Interstitial lung diseases (ILDs) constitute a diverse and complex spectrum of parenchymal lung disorders characterized by varying degrees of inflammation, fibrosis and architectural distortion of the pulmonary interstitium, ultimately culminating in progressive impairment of gaseous exchange and respiratory function. Among these, fibrotic phenotypes are particularly associated with relentless clinical deterioration, heightened symptom burden and adverse prognostic outcomes [1, 2]. While resting hypoxemia has traditionally been regarded as a hallmark of advanced disease, contemporary evidence increasingly underscores the clinical relevance of nocturnal hypoxemia, which may manifest even in patients with relatively preserved daytime oxygenation and often remains clinically under-recognized [1]. The pathophysiological basis of nocturnal oxygen desaturation in ILD is multifactorial, encompassing diminished pulmonary compliance, reduced diffusing capacity, ventilation-perfusion mismatch and alterations in central ventilatory control during sleep. These abnormalities are further accentuated during rapid eye movement (REM) sleep, wherein physiological reductions in ventilatory drive predispose patients to episodic hypoxemia and sleep fragmentation [1, 3]. Importantly, nocturnal hypoxemia has been independently associated with accelerated disease progression, worsening symptomatology and increased mortality in patients with progressive fibrotic ILD, thereby highlighting its potential role as both a marker and mediator of disease severity [2]. Sleep disturbances in ILD extend beyond isolated hypoxemic episodes and frequently encompass a broader spectrum of sleep-related abnormalities, including reduced sleep efficiency, altered sleep architecture and coexistent sleep- disordered breathing. The presence of overlapping conditions such as obstructive sleep apnea may further compound nocturnal desaturation and contribute to recurrent arousals, thereby impairing restorative sleep [4]. These disruptions have significant downstream consequences, as inadequate sleep quality is closely linked with daytime fatigue, diminished functional capacity and compromised health-related quality of life, all of which are increasingly recognized as critical patient-centered outcomes in chronic respiratory disease [5, 6]. Therapeutically, oxygen supplementation remains a fundamental component in the management of hypoxemic ILD, with established benefits in alleviating dyspnoeal and improving exercise tolerance. However, the application of nocturnal oxygen therapy represents a relatively underexplored domain. Emerging investigations suggest that supplemental oxygen administered during sleep may ameliorate nocturnal desaturation and favorable influence certain physiological parameters, including stabilization of oxygen saturation and attenuation of sleep-disordered breathing indices [3, 4-5]. Nevertheless, the extent to which these physiological improvements translate into meaningful enhancements in subjective sleep quality and reduction in daytime fatigue remains uncertain. A systematic synthesis of available literature reveals that, despite growing recognition of nocturnal hypoxemia as a clinically significant entity, there exists a paucity of rigorously designed studies specifically evaluating the impact of nocturnal oxygen supplementation on sleep-related outcomes and fatigue in ILD populations [1, 6]. Therefore, it is of interest to evaluate the effect of nocturnal oxygen supplementation on sleep quality and daytime fatigue in patients with interstitial lung disease.

## Materials and Methods:

The present investigation was designed as a prospective, randomized, placebo-controlled interventional study conducted among patients diagnosed with interstitial lung disease (ILD) attending a tertiary care respiratory center.

A total sample size of 228 participants was determined using the standard formula for comparative clinical studies:

n = (Zα/2 + Zβ)^2^ x 2σ^2^ / d^2^,

Where Zα/2 corresponds to the desired level of statistical significance (1.96 for 95% confidence interval), Zβ represents the power of the study (0.84 for 80% power), σ denotes the standard deviation derived from prior literature on sleep quality indices in ILD populations and d signifies the minimum clinically significant difference expected between groups. The calculated sample size was further adjusted to account for potential attrition and non-compliance, yielding a final recruitment target of 228 subjects. Eligible participants were adults with clinically and radiologically confirmed ILD who exhibited evidence of nocturnal desaturation on baseline overnight oximetry, while patients with unstable cardiopulmonary status, active infections, or previously established long-term oxygen therapy dependence were excluded to minimize confounding variables. Following enrolment, participants were randomly allocated, using a computer-generated randomization sequence, into two equal groups: an intervention arm receiving nocturnal oxygen supplementation and a control arm receiving placebo intervention in the form of sham oxygen delivered via identical nasal cannula apparatus without active oxygen flow, thereby ensuring allocation concealment and minimizing performance bias. The intervention group was administered low-flow supplemental oxygen during sleep hours, titrated to maintain peripheral oxygen saturation above clinically recommended thresholds, whereas the placebo group underwent an identical protocol without therapeutic oxygen delivery. Baseline and follow-up assessments included objective evaluation of sleep parameters through overnight polysomnography or validated sleep monitoring systems, alongside subjective assessment of sleep quality and daytime fatigue using standardized instruments such as the Pittsburgh Sleep Quality Index and fatigue severity scales. All collected data were systematically recorded and entered into a secured database and subsequently analyzed using IBM SPSS Statistics (Version 26.0, IBM Corporation, Armonk, NY, USA). Descriptive statistics were expressed as mean ± standard deviation or frequency distributions as appropriate, while inferential analysis was performed using independent t-tests, paired t-tests and chi-square tests for categorical variables. A p-value of less than 0.05 was considered statistically significant. Ethical approval for the study was obtained from the Institutional Ethics Committee and written informed consent was secured from all participants prior to inclusion, in accordance with the principles outlined in the Declaration of Helsinki.

## Results:

The present study evaluated the effect of nocturnal oxygen supplementation on sleep quality and daytime fatigue among patients with interstitial lung disease. A total of 228 participants were randomized into intervention and control groups, with complete follow-up achieved, thereby ensuring robustness of the dataset and minimizing attrition bias. As illustrated in Table 1 (see PDF), the baseline demographic and clinical parameters, including age distribution, gender ratio, body mass index, resting oxygen saturation and diffusing capacity of the lungs for carbon monoxide (DLCO), were statistically comparable between the two groups (p > 0.05). Furthermore, baseline scores for sleep quality (PSQI) and fatigue severity did not demonstrate any significant intergroup variation. This homogeneity confirms the adequacy of randomization and ensures that subsequent differences observed post-intervention can be reliably attributed to the therapeutic effect of nocturnal oxygen supplementation rather than confounding variables. A comparative evaluation of sleep-related parameters, as presented in Table 2 (see PDF), revealed a marked and statistically significant improvement in the oxygen supplementation group following the intervention period. Mean nocturnal oxygen saturation increased substantially from 88.5 ± 3.2% to 93.4 ± 2.5%, whereas only a marginal rise was observed in the control group. Similarly, the apnea-hypopnea index (AHI) demonstrated a pronounced reduction in the intervention arm, indicating a decrease in sleep-disordered breathing events. Sleep efficiency exhibited a significant upward trend in the oxygen group, reflecting improved continuity and restorative quality of sleep. Additionally, the Pittsburgh Sleep Quality Index (PSQI) scores showed a notable decline, signifying enhanced subjective sleep quality. In contrast, the control group demonstrated only minimal, clinically insignificant changes across these parameters. The intergroup differences for all sleep variables were highly significant (p < 0.001), underscoring the beneficial impact of nocturnal oxygen therapy on both objective and subjective sleep indices. The assessment of daytime outcomes, detailed in Table 3 (see PDF), further corroborated these findings. The oxygen supplementation group exhibited a substantial reduction in fatigue severity scores, indicating alleviation of daytime fatigue. Concurrently, Epworth Sleepiness Scale (ESS) scores decreased significantly, suggesting reduced daytime somnolence and improved alertness. Quality of life scores demonstrated a marked improvement in the intervention group, reflecting the broader clinical benefits of enhanced nocturnal oxygenation and sleep restoration. Conversely, the control group showed only modest improvements, which did not reach the magnitude observed in the intervention arm. The statistical analysis confirmed that these differences were highly significant (p < 0.001), reinforcing the efficacy of nocturnal oxygen supplementation in improving patient-centered outcomes.

## Discussion:

The present study was undertaken to evaluate the impact of nocturnal oxygen supplementation on sleep quality and daytime fatigue in patients with interstitial lung disease (ILD) and the findings demonstrate statistically significant improvements in nocturnal oxygenation, sleep architecture and daytime functional outcomes in the intervention group compared to placebo. These results reinforce the clinical significance of nocturnal hypoxemia as a modifiable contributor to disease burden. The baseline presence of significant nocturnal desaturation in both groups observed in this study is consistent with the systematic review by Khor *et al.* [1 which reported a high prevalence of nocturnal hypoxemia even in the absence of severe daytime hypoxia. The marked improvement in nocturnal SpO_2_ following oxygen supplementation in our study further supports the concept that targeted nocturnal correction is both feasible and clinically meaningful. In addition, the association between nocturnal hypoxemia and adverse outcomes demonstrated by Myall *et al.* [2] is indirectly reflected in our findings, where improvement in oxygenation corresponded with better sleep and fatigue outcomes, suggesting a broader physiological impact. The observed improvements in sleep efficiency, apnea-hypopnea index (AHI) and PSQI scores in the oxygen group can be explained by the underlying pathophysiological mechanisms described by Vázquez and Pérez-Padilla [3] who demonstrated that supplemental oxygen stabilizes nocturnal breathing and improves oxygenation during sleep. These findings are further corroborated by the randomized crossover trial by Han *et al.* [4] which showed that oxygen therapy significantly reduces sleep-disordered breathing events and enhances deeper sleep stages. The current study extends these observations by demonstrating that such physiological improvements translate into meaningful subjective sleep quality enhancement. Importantly, the role of comprehensive supportive care in ILD management must also be acknowledged. Holland *et al.* [5] emphasized the importance of pulmonary rehabilitation and structured patient education in improving overall patient outcomes, including symptom perception and functional status. While such interventions primarily target daytime symptoms, the present study demonstrates that nocturnal oxygen supplementation complements these strategies by specifically addressing night time hypoxemia, thereby contributing to a more holistic management approach. The significant reduction in fatigue severity and daytime sleepiness observed in the intervention group further underscores the clinical relevance of correcting nocturnal hypoxemia. This finding aligns with the systematic review and meta-analysis by Ahmadi *et al.* [6] which highlighted the role of oxygen therapy in alleviating symptom burden in chronic respiratory diseases. However, given the limited direct evidence linking oxygen therapy to fatigue outcomes, the present study provides important additional data supporting this association. The broader benefits of oxygen therapy in ILD are well documented. The improvements in quality of life and functional capacity observed in our study are consistent with the randomized controlled trial by Khor *et al.* [7] which demonstrated the efficacy of ambulatory oxygen in reducing dyspnea and improving activity tolerance. Similarly, the findings of Olson *et al.* [8] who reported that worsening dyspnea precedes oxygen prescription highlight the progressive nature of hypoxemic burden and support the rationale for earlier intervention, including nocturnal oxygen use. Alternative symptom-relief strategies, such as handheld fan therapy evaluated by Khor *et al.* [9] have shown modest benefits in alleviating breathlessness; however, these approaches do not address the underlying nocturnal hypoxemia. In contrast, oxygen supplementation directly targets the physiological derangements responsible for sleep disruption, as evidenced by the significant improvements in sleep parameters in the present study. From a patient-centered perspective, Graney *et al.* [10] highlighted the complex experiences associated with oxygen therapy, including both symptomatic benefits and psychosocial challenges. Despite these concerns, the significant improvement in quality-of-life scores observed in our study suggests that the therapeutic advantages of nocturnal oxygen may outweigh its limitations when appropriately implemented. Furthermore, early physiological evidence by Kramer *et al.* [11] demonstrated that increased oxygen availability improves systemic oxygenation, supporting the biological plausibility of the improvements observed in our cohort. The functional benefits of oxygen therapy are further reinforced by Dowman *et al.* [12] who reported improved endurance and reduced dyspnea with supplemental oxygen even in the absence of resting hypoxemia. These findings align with the present study, where improvements extended beyond nocturnal oxygenation to include daytime fatigue and sleepiness. Additionally, expert reviews by Khor *et al.* [13] and Clark *et al.* [14] advocate for individualized oxygen therapy strategies, emphasizing the need to tailor interventions based on patient-specific physiological and symptomatic profiles. Finally, variability in clinical practice highlighted by Khor *et al.* [15] reflects the absence of standardized guidelines for oxygen therapy in ILD. The present study contributes to this evolving evidence base by demonstrating that nocturnal oxygen supplementation yields significant improvements across multiple domains, including sleep quality, fatigue and overall quality of life, thereby supporting its integration into routine clinical practice. Despite these encouraging findings, certain limitations must be acknowledged. The relatively short follow-up duration may limit the assessment of long-term outcomes and although validated tools were employed, the multidimensional nature of sleep and fatigue may not be fully captured. Nevertheless, the placebo-controlled design and consistent improvements across objective and subjective measures strengthen the validity of the findings. Taken together, the present study, in alignment with existing literature [1-15] shows that nocturnal oxygen supplementation plays a pivotal role in correcting sleep-related hypoxemia and significantly improving both physiological and patient-centered outcomes in ILD the relatively short follow-up duration and single-center design may limit the generalizability and long-term applicability of the findings. Large-scale, multicentric longitudinal studies incorporating objective sleep metrics and patient-reported outcomes are warranted to establish standardized guidelines for nocturnal oxygen therapy in ILD.

## Advancement to knowledge:

Nocturnal oxygen supplementation (NOS) dramatically improves sleep-disordered breathing (SDB) and sleep structure in patients with comorbid obstructive sleep apnea (OSA), a common cause of daytime fatigue in this population, according to recent developments in the treatment of interstitial lung disease (ILD).

## Conclusion:

We show that nocturnal oxygen supplementation represents a clinically meaningful intervention in ILD, with the potential to improve sleep quality, reduce daytime fatigue and enhance overall quality of life. However, further large-scale, longitudinal studies to establish standardized guidelines for the use of nocturnal oxygen therapy are needed.
